# Algorithm for Individual Prediction of COVID-19–Related Hospitalization Based on Symptoms: Development and Implementation Study

**DOI:** 10.2196/29504

**Published:** 2021-11-15

**Authors:** Rossella Murtas, Nuccia Morici, Chiara Cogliati, Massimo Puoti, Barbara Omazzi, Walter Bergamaschi, Antonio Voza, Patrizia Rovere Querini, Giulio Stefanini, Maria Grazia Manfredi, Maria Teresa Zocchi, Andrea Mangiagalli, Carla Vittoria Brambilla, Marco Bosio, Matteo Corradin, Francesca Cortellaro, Marco Trivelli, Stefano Savonitto, Antonio Giampiero Russo

**Affiliations:** 1 Epidemiology Unit, Agency for the Protection of Health of the Metropolitan Area of Milan Milan Italy; 2 ASST Grande Ospedale Metropolitano Niguarda Milan Italy; 3 Department of Clinical Sciences and Community Health, Università degli Studi di Milano Milan Italy; 4 ASST Fatebenefratelli-Sacco, Luigi Sacco Hospital Milan Italy; 5 Università degli Studi Milano Bicocca, School of Medicine Milan Italy; 6 ASST Rhodense Milan Italy; 7 Agency for the Protection of Health of the Metropolitan Area of Milan Milan Italy; 8 IRCCS Humanitas Rozzano Italy; 9 IRCCS San Raffaele Milan Italy; 10 General Practitioners Group, Azienda Territoriale della Salute, Milan Metropolitan Area Milan Italy; 11 Ordine dei Medici Chirurghi e degli Odontoiatri di Milano Milan Italy; 12 ASST Santi Paolo and Carlo Milan Italy; 13 ASST Brianza Vimercate Italy; 14 Ospedale A. Manzoni Lecco Italy

**Keywords:** COVID-19, severe outcome, prediction, monitoring system, symptoms, risk prediction, risk, algorithms, prediction models, pandemic, digital data, health records

## Abstract

**Background:**

The COVID-19 pandemic has placed a huge strain on the health care system globally. The metropolitan area of Milan, Italy, was one of the regions most impacted by the COVID-19 pandemic worldwide. Risk prediction models developed by combining administrative databases and basic clinical data are needed to stratify individual patient risk for public health purposes.

**Objective:**

This study aims to develop a stratification tool aimed at improving COVID-19 patient management and health care organization.

**Methods:**

A predictive algorithm was developed and applied to 36,834 patients with COVID-19 in Italy between March 8 and the October 9, 2020, in order to foresee their risk of hospitalization. Exposures considered were age, sex, comorbidities, and symptoms associated with COVID-19 (eg, vomiting, cough, fever, diarrhea, myalgia, asthenia, headache, anosmia, ageusia, and dyspnea). The outcome was hospitalizations and emergency department admissions for COVID-19. Discrimination and calibration of the model were also assessed.

**Results:**

The predictive model showed a good fit for predicting COVID-19 hospitalization (C-index 0.79) and a good overall prediction accuracy (Brier score 0.14). The model was well calibrated (intercept –0.0028, slope 0.9970). Based on these results, 118,804 patients diagnosed with COVID-19 from October 25 to December 11, 2020, were stratified into low, medium, and high risk for COVID-19 severity. Among the overall study population, 67,030 (56.42%) were classified as low-risk patients; 43,886 (36.94%), as medium-risk patients; and 7888 (6.64%), as high-risk patients. In all, 89.37% (106,179/118,804) of the overall study population was being assisted at home, 9% (10,695/118,804) was hospitalized, and 1.62% (1930/118,804) died. Among those assisted at home, most people (63,983/106,179, 60.26%) were classified as low risk, whereas only 3.63% (3858/106,179) were classified at high risk. According to ordinal logistic regression, the odds ratio (OR) of being hospitalized or dead was 5.0 (95% CI 4.6-5.4) among high-risk patients and 2.7 (95% CI 2.6-2.9) among medium-risk patients, as compared to low-risk patients.

**Conclusions:**

A simple monitoring system, based on primary care data sets linked to COVID-19 testing results, hospital admissions data, and death records may assist in the proper planning and allocation of patients and resources during the ongoing COVID-19 pandemic.

## Introduction

With 85,783,178 infections and 1,855,872 deaths as of January 5, 2021 [1], the ongoing COVID-19 pandemic has put an unprecedented strain on the health care system worldwide. Three different priorities can be envisaged in order to limit the impact of virus spread: (1) social and occupational health measures to decrease the risk of an airborne spread; (2) population screening using mass testing to identify sources of infection, with subsequent isolation of those who test positive for COVID-19; and (3) more selective testing of symptomatic patients to identify those with a confirmed diagnosis of COVID-19 (as opposed to influenza-like illness), as well as those patients who will most likely need hospital admission. Although the first and second tasks pertain to health care authorities, the third is typical of primary care, provided that validated predictive algorithms are available.

General practitioners (GPs) are in the forefront of this process and should be provided with tools that have inherent clinical sense and are easy to use to facilitate quick decision-making given the overwhelming numbers of patients they are engaged with in daily clinical practice. Even though several prediction models have been developed, their predictive performance has been questioned because of their ability to be representative of the general population [2,3]. A real-world approach, using primary care data sets linked to the testing results, hospital admissions data, and death records, has been extensively developed in the British population in order to assist risk prediction of hospital admission and mortality due to COVID-19 [4]. This methodology might be informative in order to detect patients with COVID-19 and, among them, those with higher risk of requiring early hospital admission. Our working group has previously released a consensus document drawn up by hospital consultant physicians and GPs in order to stratify patients with symptoms suspected of SARS-CoV-2 infection and improve their management in appropriate “hot spot” facilities [5].

However, a comprehensive analysis in Lombardy region, Italy, that uses all data available in the administrative data set is currently lacking. This could potentially be highly useful to inform and guide treatment and vaccination campaigns. In the initial weeks of March, when the COVID-19 epidemic was growing exponentially, a predictive model was developed to stratify patient risk of dying at the individual level, according to age and the presence of comorbidities [6].

Here, we aimed to evaluate potential risk factors for hospitalization. Therefore, with the start of the second wave of COVID-19, we further implemented an algorithm to estimate, among patients with COVID-19, the risk of being admitted to the hospital with SARS-CoV-2 infection based on sex, age, COVID-19 symptoms, and comorbidities. Second, by combining the algorithms for the risk of overall mortality and that for the risk of hospitalization, we propose a stratification method (ie, low, medium, and high risk) that has been successfully implemented for patients with COVID-19.

## Methods

### Ethics Approval and Consent to Participate

This study was conducted in accordance with ethical principles based on the Declaration of Helsinki [7] and current ethical guidelines. No individual-level data were used for this study, and patients cannot be identified from aggregated data that do not contain low counts. For this reason, and in accordance with the Italian legislation, this study was not submitted for ethics approval [8].

### Study Setting

From March 8, 2020, onward, a surveillance system of the Agency for Health Protection of Metropolitan Area of Milan (ATS Milan) collected data on all residents of the territory who had either a positive or negative COVID-19 test result. A confirmed case is defined as a person with a real-time reverse-transcription polymerase chain reaction (RT-PCR) positive test result for SARS-CoV-2, irrespective of clinical signs and symptoms. In addition, GPs inputted individual patient data on the presence or absence of specific symptoms associated with COVID [9-15], namely, vomiting, cough, fever, diarrhea, myalgia, asthenia, headache, anosmia, ageusia, and dyspnea.

### Predictive Algorithm for Risk of Hospitalization Due to COVID-19

From the surveillance system developed by the ATS of Milan, we collected data on all patients with a positive test result for SARS-CoV-2 between March 8 and October 9, 2020, along with additional information reported by GPs about the presence or absence of COVID-19 symptoms.

Using the administrative discharge records from ATS Milan, all hospitalizations and emergency department admissions occurring in the 31 days before or after the date of inclusion in the cohort were also collected. Date of inclusion in the cohort was defined as the date of symptom onset for symptomatic patients, and date of first positive swab in asymptomatic patients. We decided to include asymptomatic patients because their status of having no symptoms contributed to nonhospitalization data. Hospital admissions data for COVID-19 cases were shortlisted from total hospital admissions data based on the cases with International Classification of Diseases-9 (ICD-9) [16] codes V01.82, 079.82, 480.3, V07.0, and 078.89. Individual-level comorbidity data were derived using the chronic disease administrative database of ATS Milan, according to the algorithms specified in the Regional Act X/6164 [17] and X/7655 [18] of 2017. These algorithms are based on the following databases: hospital discharge, outpatient visits and exams, exempt from copayment, and drug prescriptions.

To assess the association between COVID-19–related hospitalization and the presence of symptoms in COVID-19–positive patients, we implemented a logistic regression model adjusted for age (as a continuous variable, where each value represented an increase in age of 5 years compared to the preceding value); sex (reference category: female); and comorbidities, such as cardiovascular disease (eg, peripheral artery disease, chronic heart failure, venous disease, ischemic heart disease, valvular heart disease, and cardiomyopathy with and without arrhythmia), hypercholesterolemia, hypertension, diabetes, chronic gastrointestinal (GI) disease (eg, chronic pancreatitis, chronic hepatitis and cirrhosis, and inflammatory bowel disease), and chronic pulmonary disease (eg, respiratory failure or oxygen therapy, chronic obstructive pulmonary disease, and asthma). Results are presented as odds ratios (ORs) with 95% CIs, and estimated model parameters are reported in in the Results section. Individual predicted probabilities were calculated by reversing the logit transformation. The algorithm for the risk of being hospitalized due to COVID-19 was developed following the TRIPOD (Transparent Reporting of a Multivariable Prediction Model for Individual Prognosis or Diagnosis) guidelines [19].

A priori clinical knowledge on the associations between symptoms and hospitalization due to COVID-19 was limited. However, given the high number of events and the minimal cost represented by the collection of this information, and to maximize the expected discrimination ability based on administrative data only, it was decided to develop a full model without performing model selection using automated statistical techniques.

The validation of the algorithm was assessed internally using bootstrap resampling (1000 repetitions) to evaluate the discrimination and calibration of the model [20]. Discrimination was assessed using the C-index/area under the curve (AUC) value [21], which produces a value of 1 for ideal discrimination and a value of 0.5 for discrimination that is no better than chance. A value between 0.7 and 0.8 is considered *fair* and that between 0.8 and 0.9 is considered *good* [22]. Model calibration was evaluated by estimating calibration intercept and slope, where an intercept close to 0 and a slope close to 1 indicate *good* calibration and provided a calibration plot [23]. In addition, Brier score was evaluated to estimate overall prediction accuracy, which ranges from 0 (*perfect*) to 0.25 (*worthless*) for sensible models [20].

Validation and calibration of the model were performed using R software (version 4.0.5; R Core Team) and R package rms (version 6.2-0; F. Harrel).

### Risk Stratification Model for Patients With COVID-19

Beginning on October 25, 2020, with the start of the second wave of COVID-19 in Lombardy, we developed a surveillance and monitoring system for patients with COVID-19. Each patient was stratified as a high-, medium-, or low-risk patient for the combined outcome of hospitalization and death, according to the clinical and demographic characteristics highlighted by 2 predictive models developed by ATS Milan—the aforementioned predictive model for hospitalization and the predictive model for the overall mortality risk [6].

We thus defined the risk of a patient as follows:

- High risk: if the patient was older than 70 years and had one of the comorbidities identified by the prediction algorithm for overall mortality risk (ie, presence of neurological disorders, chronic heart failure, ischemic heart disease, valvular disease, renal failure, and neoplasm diagnosed in the last 2 years). In addition, a patient was considered at high risk if they had pneumonia within 15 days before or after the date of swabbing.
- Medium risk: if the patient was not at high risk and if the predicted probability of hospitalization was greater than or equal to 40%, as determined based on the predictive model for hospitalization. In addition, a patient was considered at medium risk if no information about their symptoms was present in the database (either because they had not registered in the portal or because although their GPs registered, they did not enter any symptoms).
- Low risk: if the patient was not at high or medium risk, was asymptomatic, or had a predicted probability of hospitalization lesser than 40%.

Considering potential misclassification, we decided to use 40% as a probability cutoff for prediction, instead of the usual 50% used in logistic models [24]; this allowed us to include a larger number of patients in the medium-risk category. We thus used the prediction algorithm for overall mortality risk [6] to define high-risk patients and the aforementioned predictive model for hospitalization for the definition of medium- and low-risk patients. Individual predicted probabilities for hospitalization were calculated for each patient according to the estimated model parameters (Table 1), as well as demographic and clinical characteristics. Clinical characteristics (ie, comorbidities) were derived, as described above, using the chronic disease administrative database of ATS Milan, according to the algorithms specified in the Regional Act X/6164 [17] and X/7655 [18] of 2017, which are based on the following databases: hospital discharge, outpatient visits and exams, exempt from copayment, and drug prescriptions. Symptom data were obtained, as described above*,* from the information inputted by GPs.

**Table 1:** Demographic and clinical characteristics of training and validation sets and risk factors for COVID-19–related hospitalization. Data sourced from the surveillance system developed by the Agency for Health Protection of Metropolitan Area of Milan, which covers the provinces of Lodi and Milan in Italy, comprising swab-positive SARS-CoV-2 cases between March 8 and October 9, 2020, for which general practitioners reported the presence or absence of COVID-19 symptoms.

Characteristic		Predictive algorithm for COVID-19 hospitalization, n (%)				OR^a^ (95% CI)^b^	
		Overall (N=36,834)	Training (n=29,563)	Validation (n=7271)			
Sex, male		16,591 (45.04)	13,361 (45.20)	3230 (44.42)	2.46 (2.33-2.61)		
**Age in years**							1.12 (1.11-1.13)
	<18	3186 (8.65)	2582 (8.73)	604 (8.31)			
	18-40	6402 (17.38)	5107 (17.27)	1295 (17.81)	—^c^		
	40-70	13,943 (37.85)	11,252 (38.06)	2691 (37.01)	—		
	≥70	13,303 (36.12)	10,622 (35.93)	2681 (36.87)	—		
Outcome = yes		8069 (21.91)	6468 (21.88)	1601 (22.02)	—		
Asymptomatic		4475 (12.15)	3589 (12.14)	886 (12.19)	—		
**Symptoms** **= yes**							
	Vomiting	791 (2.15)	639 (2.16)	152 (2.09)	1.43 (1.16-1.75)		
	Cough	9889 (26.85)	7954 (26.91)	1935 (26.61)	1.23 (1.15-1.32)		
	Fever	18,747 (50.9)	15,092 (51.05)	3655 (50.27)	1.84 (1.73-1.96)		
	Diarrhea	2416 (6.56)	1939 (6.56)	477 (6.56)	0.85 (0.74-0.97)		
	Myalgia	3634 (9.87)	2922 (9.88)	712 (9.79)	0.50 (0.44-0.57)		
	Asthenia	6383 (17.33)	5101 (17.25)	1282 (17.63)	0.57 (0.52-0.63)		
	Headache	3201 (8.69)	2566 (8.68)	635 (8.73)	0.59 (0.51-0.68)		
	Anosmia	1874 (5.09)	1494 (5.05)	380 (5.23)	0.18 (0.13-0.24)		
	Ageusia	708 (1.92)	573 (1.94)	135 (1.86)	1.04 (0.69-1.57)		
	Dyspnea	7487 (20.33)	6013 (20.34)	1474 (20.27)	3.95 (3.72-4.21)		
**Comorbidities = yes**							
	Cardiovascular disease	7140 (19.38)	5676 (19.20)	1464 (20.13)	0.86 (0.79-0.92)		
	Hypercholesterolemia	4183 (11.36)	3345 (11.31)	838 (11.53)	1.32 (1.21-1.43)		
	Hypertension	12,167 (33.03)	9773 (33.06)	2394 (32.93)	1.40 (1.31-1.51)		
	Diabetes	3742 (10.16)	3036 (10.27)	706 (9.71)	1.39 (1.28-1.51)		
	GI^d^ disease	1085 (2.95)	869 (2.94)	216 (2.97)	1.34 (1.16-1.54)		
	Pulmonary disease	2745 (7.45)	2179 (7.37)	566 (7.78)	1.28 (1.17-1.41)		

^a^OR: odds ratio.

^b^OR and corresponding 95% CI values were calculated from a multivariate logistic model, including age (5-year age classes), sex (reference category: female), COVID-19 symptoms (eg, vomiting, cough, fever, diarrhea, myalgia, asthenia, headache, anosmia, ageusia, and dyspnea), and comorbidities (eg, cardiovascular disease, hypercholesterolemia, hypertension, diabetes, gastrointestinal disease, and pulmonary disease).

^c^Not available.

^d^GI: gastrointestinal.

The system granted a telephone call by a trained operator who assessed the patient’s state of health and, if necessary, gave advice to the patient to visit the hospital or emergency department. For this purpose, ATS Milan trained an internal call center as well as external operators, who received a set of patients to be contacted on a daily basis. Each call center received a number of patients in line with its capacity (based on the number of operators, staff roasters, etc). This number was decided by each call center during the implementation of the system, and eventually updated during the epidemic according to staff modifications. Given the huge number of positive cases and the limited capacity of the call centers, we decided to send patients to surveillance in order of priority: first high-risk patients, followed by medium- and low-risk patients.

The second part of this study intends to present the results of this monitoring system from October 25 to December 11, 2020. To measure the association between patient stratification as high, medium, and low risk and actual severity of COVID-19, we used ordinal (cumulative) logistic models. Severity of COVID-19 was defined as an ordinal outcome equal to 0 if treated at home (home-treated), equal to 1 if hospitalized, and equal to 2 if deceased. The models were adjusted for sex, age, and comorbidities (cardiovascular disease, hypercholesterolemia, hypertension, diabetes, chronic gastrointestinal disease, and chronic pulmonary disease). Results are presented as odds ratios (ORs) with 95% CI values. ORs for the ordinal logistic model are interpreted in their cumulative formulation, that is, the odds of deceased versus the combined categories of hospitalized and home-treated patients, as well as of the combined categories of deceased and hospitalized versus home-treated patients. The analyses were performed using SAS software (version 9.4; SAS Institute Inc).

### Availability of Data and Materials

Data are not publicly available because they are owned by ATS Milan and cannot be distributed to third parties.

## Results

### Study Cohort Used for the Predictive Algorithm for Risk of COVID-19–Related Hospitalization

From March 8, 2020, to October 9, 2020, we collected data of 36,834 patients with a positive test result for COVID-19 (demographic and clinical characteristics are reported in Table 1), for which the patients’ GPs reported the presence or absence of COVID-19 symptoms. Among these patients, 8069 (22%) were hospitalized or admitted to an emergency department with a COVID-19 diagnosis. Fever, cough, and dyspnea were the most common symptoms, reported by more than 20% of the cohort, whereas 12.15% (4475/36,834) of the cohort comprised asymptomatic COVID-19 cases. In this cohort (N=36,834), 19.38% (n=7140) had cardiovascular diseases, 11.36% (n=4183) had hypercholesterolemia, 33.03% (n=12,167) had hypertension, 10.16% (n=3742) had diabetes, 2.95% (n=1085) had GI disease, and 7% (n=2745) had pulmonary disease.

Table 1 presents the OR and corresponding 95% CI values from the logistic regression model estimating the risk of COVID-19 hospitalization. The likelihood of being hospitalized for COVID-19 was higher among older patients, with increasing odds of 12% for an increase in age-class (OR 1.12, 95% CI 1.11-1.13), male patients with OR 2.46 (95% CI 2.33-2.61 vs female patients). Vomit, cough, fever, and dyspnea were statistically significant risk factors for COVID-19 hospitalization, whereas no association was found for ageusia. On the other hand, diarrhea, myalgia, asthenia, headache, and anosmia showed a negative association with the risk of COVID-19–related hospitalization.

This algorithm produced a C-index of 0.79, which suggest a *fair* and *almost good* discriminator ability to predict COVID-19 hospitalization. This model had good overall prediction accuracy (Brier score 0.14) and was well calibrated (intercept –0.0028, slope 0.9970; see Multimedia Appendix 1 for calibration plot).

We acquired BMI information at diagnosis for a subset of the general cohort (4586/36,834, 12.45%): 9.62% (441/4586) were underweight (BMI<18.5), 53.1% (2435/4586) were normal weight (BMI 18.5-24.9), 26.65% (1222/4586) were overweight (BMI 25-29.9), and 10.64% (488/4586) were obese (BMI ≥30). According to a logistic regression model adjusted for age and sex, BMI (continuous variable) was associated with a higher risk of being hospitalized (OR 1.05, 95% CI 1.03-1.08), and overweight and obese were associated with a high risk of being hospitalized compared to normal weight (OR 1.4, 95% CI 1.04-1.8, and OR 1.9, 95% CI 1.3-2.6, respectively). In the subset with nonmissing BMI information, we evaluated the discrimination ability of the same model described previously, including BMI (continuous variable), which produced a c-index of 0.89.

### Results of the Epidemiological Monitoring System

**Figure 1:**
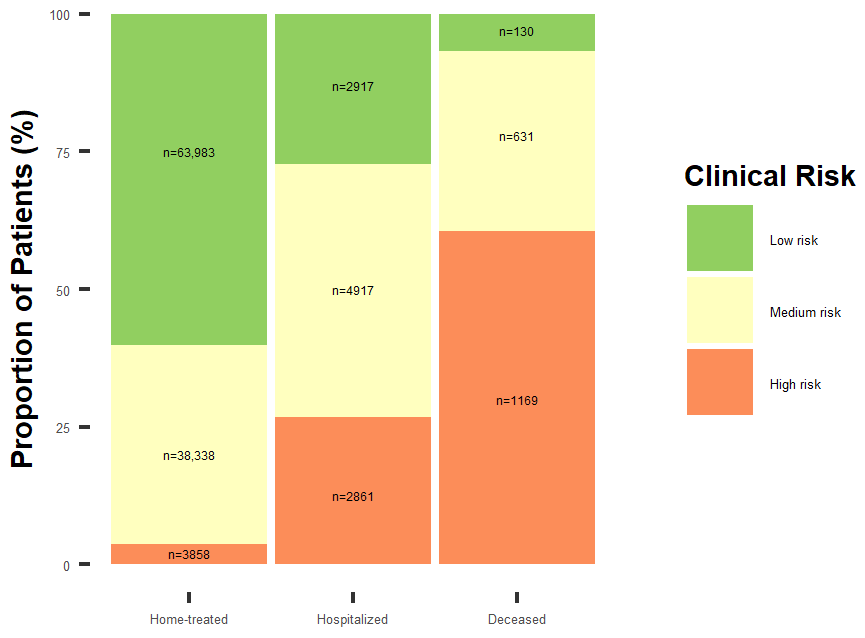
Stratification of study patients by clinical risk and health status, based on results from the monitoring system developed during the second wave of COVID-19 by the Agency for Health Protection of Metropolitan Area of Milan (data updated on December 11, 2020).

Beginning on October 25, 2020, with the start of the second wave of COVID-19 in Lombardy, we developed a surveillance and monitoring system for COVID-19 patients, stratifying (up to December 11, 2020) 118,804 COVID-19 cases into high-, medium-, and low-risk patients. Among these, 63,816 (53.72%) were actually included in the surveillance system and 39,167 (32.97%) were contacted by trained call center operators. Of the overall population, 67,030 (56.42%) were defined as low risk; 43,886 (36.94%), at medium risk; and 7888 (6.64%), as high risk. As of December 11, 2020, 89.37% (106,179/118,804) of the overall population was assisted at home, 9% (10,695/118,804) was hospitalized, and 1.62% (1930/118,804) had died. Among those assisted at home, the majority of patients (67030/118,804, 56.42%) were classified as low risk, whereas only 6.64% (7888/118,804) were classified as high risk (Figure 1). Among those hospitalized, 45.97% (4917/10,695) were classified to be at medium risk, and 26.75% (2861/10,695) were classified to be at high risk. Among the deceased, 60.57% (1169/1930) were classified to be at high risk and 6.74% (130/1930), at low risk.

According to the ordinal logistic model adjusted for age, sex, and comorbidities, we found statistically significant associations between having a severe outcome (ie, hospitalized or deceased) and the proposed stratification. Patients classified at high risk had an OR of 5.0 (95% CI 4.6-5.4) of having a worse outcome compared to low-risk patients. Patients classified at medium risk had an OR of 2.7 (95% CI 2.6-2.9) of having a worse outcome compared to low-risk patients.

## Discussion

### Principal Findings

In this study, we developed a risk prediction model for COVID-19 hospitalization using age, sex, symptoms, and comorbidities. The model showed a good discriminator ability that could be sensibly improved by including BMI information in the model prediction. However, this model had a good discriminative capability, especially considering that predictors were derived from administrative data [25]. The model highlighted vomit, cough, fever, and dyspnea as statistically significant risk factors for COVID-19 hospitalization. However, we found no association for ageusia, which was probably underestimated in the first wave of the epidemic. The main result of our research, performed using a population-based approach, is the development of a simple and robust stratification tool aimed at improving COVID-19 patient management and health care organization. This tool was constructed combining 2 predictive models developed by ATS Milan: the predictive model for hospitalization and the predictive model for overall mortality risk [6]. Using these predictive models, a stratification tool was easily generated with a close relationship between patient stratification and the health status. Among patients who were managed at home, only 3.63% (3858/106,179) were at high risk. In contrast, among those who died, 60.57% (1169/1930) were at high risk, and only 6.74% (130/1930) were at low risk. Results suggest that patients classified to be at high and medium risk were at higher risk of having a worse outcome than those classified to be as low risk. Most importantly, these data confirm the relevance of an integrated approach in patient management and the leading role of GPs surveillance in improving outcomes.

Since the first COVID-19 outbreak, there was a need to obtain risk stratification tools to assist clinicians in their decision-making, considering the limited resources available. With the spread of the pandemic, the strategy of focusing on an integrated approach of care became increasingly important in order to avoid the collapse of the hospital system and to preserve a high level of care for most critical COVID-19 cases, as well as for cardiovascular or oncological cases. The Metropolitan area of Milan was one of the most impacted areas worldwide, with coronary care units and surgical operating rooms converted to general intensive care units for patients with COVID-19 requiring high-dependency care, and noninvasive ventilation made available in converted internal medicine and infectious disease units. However, the high rate of patients who were mildly symptomatic or asymptomatic fosters the idea that, in most cases, the disease could be controlled by closely monitoring its course.

Since then, an approach based on record linkage between different health registries of COVID-19 testing results along with the implementation of a surveillance system has emerged as a practical and powerful option to balance the health resources and targeting interventions. In this study, we suggest an algorithm to predict the risk of COVID-19–related hospitalization that would be fundamental to the early implementation of measures of prevention and containment in the upcoming months.

Interventions that prevent COVID-19 progression can be expected to reduce the morbidity and mortality of infection, frequency of hospitalization, and current unbearable strain on health system. Monoclonal antibodies and hyperimmune plasma used early in outpatients have shown efficacy in reducing viral load and nasopharyngeal shedding, respectively [26,27], which are related to disease severity and hospitalization rate [28]. Moreover, such treatments are expensive and logistically challenging, but they may encourage early and rapid testing of persons at high risk for SARS-CoV-2 infection and use of algorithms to identify at diagnosis those who are at risk of hospitalization and death. By using the proposed algorithm, it was possible to identify 7.888 high-risk patients out of a total of 118.804 patients with a COVID-19 diagnosis (6.63%) during the second wave. In all, 1169 (15%) patients died, and 2861 (36%) were hospitalized. Therefore, the use of this algorithm could also be applied in order to improve the cost benefit of early antiviral treatments in patients with COVID-19.

### Strengths and Limitations

Our work has several strengths, including the prospective recording of data and outcome, with minimal risk of ascertainment and performance bias, appropriate record linkage, along with validation in larger and different temporal frames. Finally, the model was based on variables readily available for each GP, who are the leading figures in providing care to the patients and primarily driving their clinical course. Thus, the described epidemiological surveillance system could launch a workflow for improving patient management well in the context of the COVID-19 pandemic, thereby informing the management of chronic conditions.

A major limitation of this study is the absence of a granular assessment of other prognostically important variables (eg, chronic kidney disease, tobacco use, and BMI), which have been implemented in other algorithms [4,29]. However, the variables included are the most easily available and collected in an administrative data set. Accordingly, recent systematic and critical reviews of modelling techniques have reported that predictions obtained using more complex models may not provide better information or be more reliable than those obtained using a simpler model [30]. Another limitation is the lack on BMI information that, in a subset of the overall population, sensitively improved the discrimination ability of the model. In addition, we investigated the effect of age on the risk of hospitalization for COVID-19 as a mere confounder in the exposure-outcome relationship. Further work has to be done in order to consider the possible differences in age-related symptoms observed after SARS-CoV-2 infection [31].

### Conclusions

In conclusion, the predictive algorithms implemented and the ensuing stratification of patients with COVID-19 provided an accurate assessment of patients’ prognosis, with a good calibration of the predicted risk and an inherent clinical sense of the stratification tool. If systematically implemented, it will allow for a prompt identification of the most appropriate pathway of care for each patient affected by COVID-19.

## Appendix

Multimedia Appendix 1 Calibration plot of the multivariable logistic regression model predicting hospitalization risk due to COVID-19.

## Abbreviations

ATS Milan: Agency for Health Protection of Metropolitan Area of Milan
AUC: area under the curve
GI: gastrointestinal
GP: general practitioner
ICD: International Classification of Diseases
OR: odds ratio
RT-PCR: reverse-transcription polymerase chain reaction
SARS-CoV-2: severe acute respiratory syndrome–coronavirus-2
TRIPOD: Transparent Reporting of a Multivariable Prediction Model for Individual Prognosis or Diagnosis
