# Supplementary figures and images for: Algorithm for Individual Prediction of COVID-19–Related Hospitalization Based on Symptoms: Development and Implementation Study
Source: JMIR Public Health Surveill. 2021 Nov 15;7(11):e29504. doi: 10.2196/29504 (PMC8594734; doi:10.2196/29504)

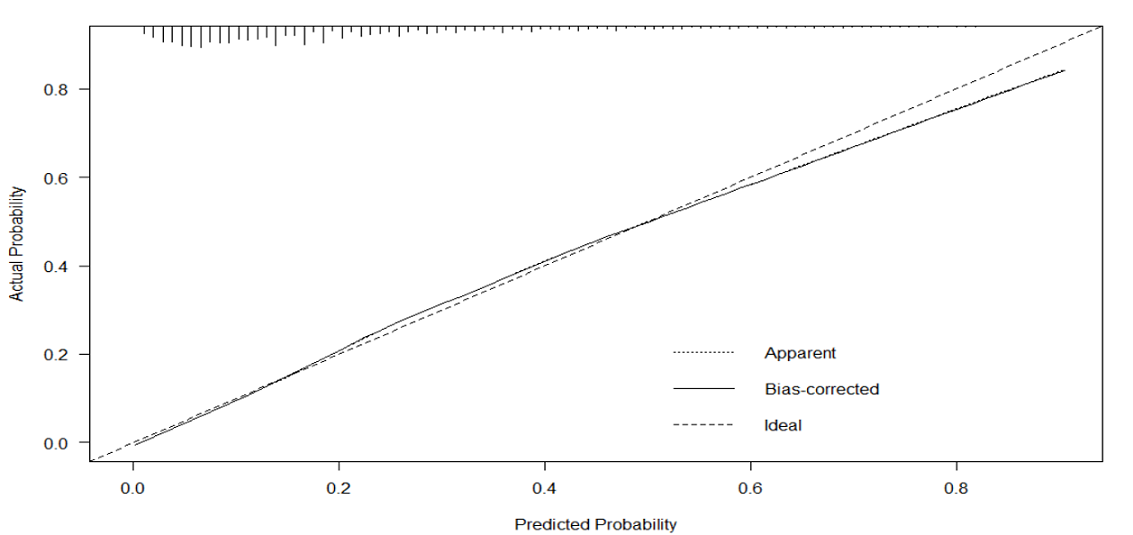

Supplement: Multimedia Appendix 1 [file publichealth_v7i11e29504_app1.png]
